# Yishenyangsui granule for degenerative cervical myelopathy: a randomized, double-blind, placebo-controlled trial with long-term follow-up

**DOI:** 10.3389/fphar.2025.1542231

**Published:** 2025-01-31

**Authors:** He Yin, Xin Chen, Zhiwei Liu, Bo Xu, Zhefeng Jin, Yan Liu, Baoyu Qi, Bin Tang, Ping Wang, Fanping Xu, Xu Wei, Jie Yu, Liguo Zhu

**Affiliations:** ^1^ Wangjing Hospital, China Academy of Chinese Medical Sciences, Beijing, China; ^2^ Spine Department, Zhangjiakou Hospital of Traditional Chinese Medicine, Zhangjiakou City, Hebei, China; ^3^ Key Laboratory of Chinese Internal Medicine of Ministry of Education, Beijing University of Chinese Medicine Affiliated Dongzhimen Hospital, Beijing, China; ^4^ First Teaching Hospital of Tianjin University of Traditional Chinese Medicine, Tianjin, China; ^5^ Spine Department, Beijing Hospital of Traditional Chinese Medicine, Beijing, China; ^6^ Beijing Key Laboratory of Manipulative Technique, Beijing, China

**Keywords:** degenerative cervical myelopathy, non-surgical treatment, Chinese botanical drugs, long-term follow-up, cervical spondylotic myelopathy

## Abstract

**Objective:**

This randomized controlled trial aims to evaluate the efficacy and safety of Yishenyangsui granule for treating Degenerative Cervical Myelopathy.

**Materials and methods:**

A randomized, double-blind, placebo-controlled clinical trial was conducted with 152 participants recruited from three centers and randomly assigned to receive either Yishenyangsui granule or placebo. The Japanese Orthopaedic Association (JOA) score and Neck Disability Index (NDI) score were evaluated for 32 weeks. Patient-reported outcomes including surgical treatment data, re-treatment data, and patient-reported condition were collected for long-term follow-up. This trial was approved by the ethics committee of WangJing Hospital of China Academy of Chinese Medical Sciences (WJEC-KT-2016-004-P001) and was registered at the Chinese Clinical Trials Registry (ChiCTR-INR-16009723) on 03 November 2016 (Check out at https://www.chictr.org.cn/indexEN.html for a more comprehensive overview).

**Results:**

The results showed that the improvement in JOA score at week 8 was significantly better in the Yishenyangsui granule group than in the placebo group (1.47 vs. 0.43; P < 0.001). Furthermore, improvements in motor function of upper/lower extremities, sensory function of upper extremities, reading ability, and recreation domain scores were also significantly superior in the Yishenyangsui granule group compared to the placebo group (P < 0.05). Long-term follow-up outcomes revealed no statistical differences between groups regarding surgical treatment data or patient-reported condition (P > 0.05). However, there was a significant difference detected in re-treatment data between groups with a lower rate observed among those receiving Yishenyangsui granule compared to those receiving placebo [25 (43.10%) vs. 40 (68.97%); P = 0.033], indicating its effectiveness for treating mild-to-moderate Degenerative Cervical Myelopathy.

**Conclusion:**

Yishenyangsui granule was effective in treating mild to moderate Degenerative Cervical Myelopathy. The participants have improved long-term outcomes.

**Clinical Trials Registration:**

https://www.chictr.org.cn/indexEN.html, identifier ChiCTR-INR-16009723.

## 1 Introduction

Degenerative cervical myelopathy (DCM) is a chronic non-traumatic disease of the spinal cord that is closely related to aging and caused by degenerative changes in the ligaments and osteochondral metabolites of the cervical spine (Theodore, 2020; Marie-Hardy and Pascal-Moussellard, 2021; Grodzinski et al., 2023). There are approximately 605 cases of clinical DCM per million people in North America (Tetreault et al., 2015). Progression of DCM could result in irreversible spinal cord damage and extremity disability and thus need an expensive operation as treatment (more than USD 2 billion per year) (Ghogawala et al., 2021; Milligan et al., 2019). The pathogenesis of DCM encompasses a multifaceted interplay of static and dynamic factors (Tu et al., 2021; Moghaddamjou et al., 2020). These factors collectively predispose to spinal cord injury (SCI), compromise the integrity of the spinal cord’s microvascular system, elicit an apoptosis cascade, and exacerbate the progression of DCM (Tran et al., 2018; Badhiwala et al., 2020). Hence, research on DCM-induced spinal cord injury focuses on the preservation of residual neuronal function and the facilitation of neuronal repair, which pose considerable challenges (Tanabe et al., 2011).

Non-surgical interventions also referred to as conservative treatments, presently employed in the management of DCM encompass physical therapy, spinal injections, immobilization via collars, and cervical traction (Kato and Fehlings, 2016; Fehlings et al., 2017; Davies et al., 2024). Compared to other non-surgical treatments, YSYS Granules offers a potential advantage by addressing both the underlying pathology and symptomatic relief of cervical myelopathy through a holistic approach. As a botanical drug, it is designed to promote neuroprotection, improve blood circulation, and enhance tissue repair, potentially offering benefits in both the mild and moderate stages of the condition (Bo-wen et al., 2022). Furthermore, its multi-target mechanism may complement other conservative therapies, providing an integrative option that combines efficacy with a favorable safety profile, particularly for patients seeking alternatives to pharmacological or physical interventions. According to the World Federation of Neurosurgical Societies Spine Committee, nonsurgical treatment is an important option for patients with mild to moderate DCM (Zileli et al., 2019) and, according to the guideline proposed by AO Spine and the Cervical Spine Research Society, nonsurgical treatment is also a feasible recommendation for treating mild DCM (Fehlings et al., 2017). However, evidence supporting the effectiveness of non-surgical interventions for DCM is limited. Although non-surgical treatment is recommended, its benefits and long-term results are still unclear due to lack of evidence, so a randomized, double-blind, placebo-controlled trial of conservative treatment is necessary (Wu et al., 2016).

The application of Traditional Chinese Medicine (TCM) for DCM has been extensively practiced, particularly in Asian countries. From the perspective of traditional Chinese medicine, DCM is caused by “deficiency” and “stasis” (Wen, 2022). Based on the pathophysiological mechanism of DCM and the characteristics of traditional Chinese medicine, combined with long-term clinical experience, the research team designed and created the Yishen Yangsui Granule (YSYS group) based on the classic traditional Chinese medicine Granule Dihuang Yinzi, which mainly consists of *Gynochthodes officinalis* (F.C. How) Razafim. and B. Bremer (9 g), *Rehmannia glutinosa* (Gaertn.) DC. (12 g), *Paeonia lactiflora* Pall. (12 g), *Astragalus membranaceus* Fisch. Ex Bunge (15 g), *Cinnamomum burmannii* (Nees and T. Nees) Blume (6 g), *Salvia miltiorrhiza* var. Miltiorrhiza (9 g), *Euonymus alatus* (Thunb.) Siebold (12 g), *Notopterygium incisum* Ting ex H.T. Chang (6 g), and *Cervus nippon* Temminck (12 g) (the plant names have been annotated by http://mpns.kew.org/mpns-portal/ on September. 25th, 2024).

Traditionally, the YSYS group is often thought to promote nerve health and reduce secondary damage (Bo-wen et al., 2022). In the initial phase of clinical randomized controlled trials, the research team corroborated the notable efficacy of the YSYS group in ameliorating the functionality of mild to moderate DCM patients (Zhiwei et al., 2022). However, its effect on DCM remains uncertain because of poor study designs and the lack of patient-reported long-term outcomes in previous studies. The purposes of this study were to estimate the efficacy of Yishenyangsui granules for improving functional ability and to explore the patient-reported long-term outcomes of treatment for mild to moderate DCM.

## 2 Materials and methods

### 2.1 Study design

This multicentre, parallel groups randomized, double-blind, placebo-controlled trial was conducted at 3 hospitals in China. This trial was approved by the institutional review board at each site. Written informed consent was obtained from all participants. This trial was approved by the ethics committee of Wang Jing Hospital of China Academy of Chinese Medical Sciences (WJEC-KT-2016-004-P001) and was registered at the Chinese Clinical Trials Registry (ChiCTR-INR-16009723) on 03 November 2016 (Check out at https://www.chictr.org.cn/indexEN.html for a more comprehensive overview). The study followed the CONSORT Extension for Chinese Botanical Drugal Medicine Formula 2017 (CONSORT CHM 2017) reporting guideline (Cheng et al., 2017).

### 2.2 Participants

#### 2.2.1 Inclusion criteria

Volunteers were recruited via newspapers, websites, and hospital posters. Participants who met the following criteria were included:

(1) Meet the above diagnostic criteria.
(2) Age from 40 to 70 years old.
(3) Has signed informed consent and explicitly refused surgical treatment.

#### 2.2.2 Exclusion criteria

The exclusion criteria were as follows:

(1) Diagnosed with cervical tumors, tuberculosis, or osteomyelitis.
(2) Spinal injury, fracture, or dislocation.
(3) Suffering from severe heart, lung, brain, liver, kidney, hematological diseases, or mental disorders.
(4) Severe skin damage, infection, or dermatological diseases in the treatment area.
(5) History of cervical spine surgery or congenital cervical deformities.
(6) Clear surgical indications, including Frankel grade above D, incontinence, MRI showing spinal cord compression ratio <0.4, ineffective conservative treatment for over 3 months, and progressively worsening symptoms.

#### 2.2.3 Withdrawal criteria

(1) Poor compliance by participants, including failure to follow the prescribed dosage or attend fewer than three treatment sessions.
(2) Loss to follow-up for any reason.
(3) Participant voluntarily withdraws from the clinical trial and informs the supervising doctor.
(4) Participants meeting any of the above criteria will be classified as withdrawal cases.

#### 2.2.4 Termination criteria

(1) Severe adverse events (life-threatening or significantly impairing daily life or work) that make continued treatment unfeasible.
(2) Complete symptom resolution during the treatment period, leading to treatment discontinuation.
(3) Severe and persistent allergic reactions necessitating the cessation of study treatment.
(4) Progressive symptom worsening during treatment, including incomplete paralysis or incontinence, meeting Frankel grade above D. These cases should terminate the study and consider surgical intervention.

### 2.3 Randomization and masking

Participants were randomly allocated to receive either a Yishenyangsui granule or a placebo. The randomization sequence was generated by an online central randomization system with a 1:1 allocation ratio. The treatment assignments, data entry, online randomization system, and scheme implementation were supervised by researchers at a tertiary service center.

Group allocation was concealed from the participants, therapists delivering the intervention, outcome assessors, and statisticians.

### 2.4 Preparation of concentrated granules

Yishenyangsui granules were prepared from 93 g of the following botanical drug substances: Bajitian, Morindae Officinalis Radix [Gynochthodes officinalis (F.C. How) Razafim. and B. Bremer], 9 g; Dihuang, Rehmanniae Radix [Rehmannia glutinosa (Gaertn.) DC.], 12 g; Lujiaoshuang, Cervi Cornu Degelatinatum [Cervus nippon Temminck], 12 g (Lujiaoshuang is obtained from the natural shedding of artificially raised sika deer after horn ossification. It is a renewable resource and does not affect the sika deer population.); Baishao, Paeoniae Radix Alba [Paeonia lactiflora Pall.], 12 g; Huangqi, Astragali Radix [Astragalus membranaceous Fisch. ex Bunge], 15 g; Guizhi, Cinnamomi Ramulus [Cinnamomum burmanni (Nees and T. Nees) Blume], 6 g; Danshen, Salviae Miltiorrhizae Radix et Rhizoma [Salvia miltiorrhiza var. Miltiorrhiza], 9 g; Guijianyu, Euonymus Alatus [Euonymus alatus (Thunb.) Siebold] 12 g; Qianghuo, Notopterygii Rhizoma et Radix [Notopterygium incisum Ting ex H.T.Chang], 6 g. These botanical drugs were decocted and then filtered to prepare a Granule solution. The Granule solution was concentrated and granulated with a recipient to form 9.15 g of granules. All botanical drugs were purchased and decocted by Sichuan New-Green Pharmaceutical Technology Development Co., Ltd. The plant name has been checked with http://mpns.kew.org/mpns-portal/ on September 25th, 2024 (Supplementary Table S1).

The placebo contained 85.0% maltodextrin, 5.0% caramel coloring, and a 10.0% mixture of Yishenyangsui granules. Yishenyangsui granules and the placebo were produced by the uniform quality control criteria by Sichuan New-Green Pharmaceutical Technology Development Co., Ltd. The entire study was conducted using the same batch of granules. Both the Yishenyangsui granule and the placebo were sealed in an aluminum foil sachet and were identical in outward appearance. The Yishenyangsui granules and the placebo were stored in an electronic cabinet at a temperature of 4°C and a humidity between 20% and 30%. The electronic cabinet was displayed in a special room for clinical trials and was managed by a qualified clinical pharmacist.

### 2.5 Ultra-high performance liquid chromatography sample preparation

For ultra ultra-high performance liquid chromatography (UHPLC) analysis, sample pretreatment method: accurate water decoction (2 mL), addition of methanol (8 mL), high-speed mixing to extract (3 min), extract obtained (1 mL), 14,000 rpm speed centrifugation (10 min), supernatant obtained.

### 2.6 UHPLC analysis

A Waters Acquity-Class ultra ultrahigh-performance liquid chromatography (UHPLC) tandem Waters Synapt G2-SiQ-TOF high-resolution mass spectrometry system was used to identify major metabolites in the YSYS group. The UHPLC fingerprint of the YSYS group tablet is shown in Supplementary Figure S1, and the HPLC test method is included in Supplementary Table S2.

### 2.7 Intervention

Participants in the Yishenyangsui granule group (YSYS group) received Yishenyangsui granules (1 bag orally 2 times daily for 8 weeks). Participants in the placebo group received the placebo (1 bag orally 2 times daily for 8 weeks).

In the original scheme, participants were designated to receive treatments for 8 weeks, and then functional ability was assessed with the JOA scale and NDI scale (stage I) for the following 32 weeks. The long-term follow-up was designed to be performed every 1.5 years for a total of 5 years (stage Ⅱ).

According to the suggestions of the experts at the prestudy meeting, all participants were to receive mecobalamin tablets (Eisai China Holdings Ltd.) as routine treatment following the same administration scheme (0.5 mg orally 3 times daily for 8 weeks).

### 2.8 Outcomes

The primary outcome was the JOA score (Okada et al., 1993) at week 8. The JOA scale is an investigator-administered tool used to evaluate neurological function in patients with DCM. A score of 17 reflects no neurological deficit, whereas a lower score indicates a greater degree of disability and functional impairment.

The secondary outcomes were the JOA score at week 32 and the NDI (Neck Disability Index) scores at week 8 and week 32 (Vernon and Mior, 1991). The NDI scale is a composite of 10 items. Each item is scored out of 5 for a maximum total score of 50. A higher score indicates more severe dysfunction. The domain scores of the JOA scale, including motor function of the upper extremities and lower extremities, sensory function of the upper extremities, lower extremities and trunk, and bladder function, were analyzed as the secondary outcomes. The domain scores of the NDI scale, including neck pain intensity, personal care, lifting, reading, headache, concentration, work, sleeping, driving, and recreation, were also analyzed as the secondary outcomes.

Due to the impact of the coronavirus (COVID-19) pandemic, the first 1.5-year follow-up was missed, and we had to terminate the trial early and took the 3rd year patient-reported outcomes as the long-term follow-up results (stage Ⅱ) according to the long follow-up criteria guidelines (Fehlings et al., 2017) and the suggestions of the experts. During the stage II follow-up, patient-reported outcomes were collected by telephone interview, which included surgical treatment data, retreatment data, and patient-reported condition.

Adverse events were documented by participants and outcome assessors on a form throughout the trial. Based on their potential association with the ingestion of medication, adverse events were categorized by outcome assessors and related specialists as either treatment-related or nontreatment-related within 24 h of occurrence.

### 2.9 Statistical analysis

Based on the unpublished pilot data, a sample size of 152 participants was estimated to provide 90% power to detect a between-group difference of 2 points in the JOA score (Tetreault et al., 2016), assuming a standard deviation of 2.89, at the two-sided 0.05 significance level and a dropout rate of approximately 20%. A 2-point decrease in the total score was identified by the receiver operating characteristic curve as the optimal threshold, with high sensitivity, specificity, and discriminative ability to differentiate clinically important improvement (Tetreault et al., 2016).

The primary outcome (the JOA score at week 8) was analyzed using repeated measures of variance analysis. The secondary outcomes (the JOA score at week 32, the NDI score at week 8 and week 32, and the domain scores of the JOA scale and NDI scale at week 8 and week 32) were also analyzed with repeated measures analysis of variance. Retreatment data and patient-reported conditions were analyzed using Fisher’s exact test. Surgical treatment data (time-to-event outcome) were assessed using Kaplan‒Meier curves and were compared using the log-rank test.

All statistical analyses were performed according to the intention-to-treat principle using SPSS version 24.0, with a 2-sided *P* value less than 0.05 was considered to be significant. No adjustment was made for multiple comparisons; therefore, secondary outcomes should be interpreted as exploratory.

## 3 Results

**FIGURE 1:**
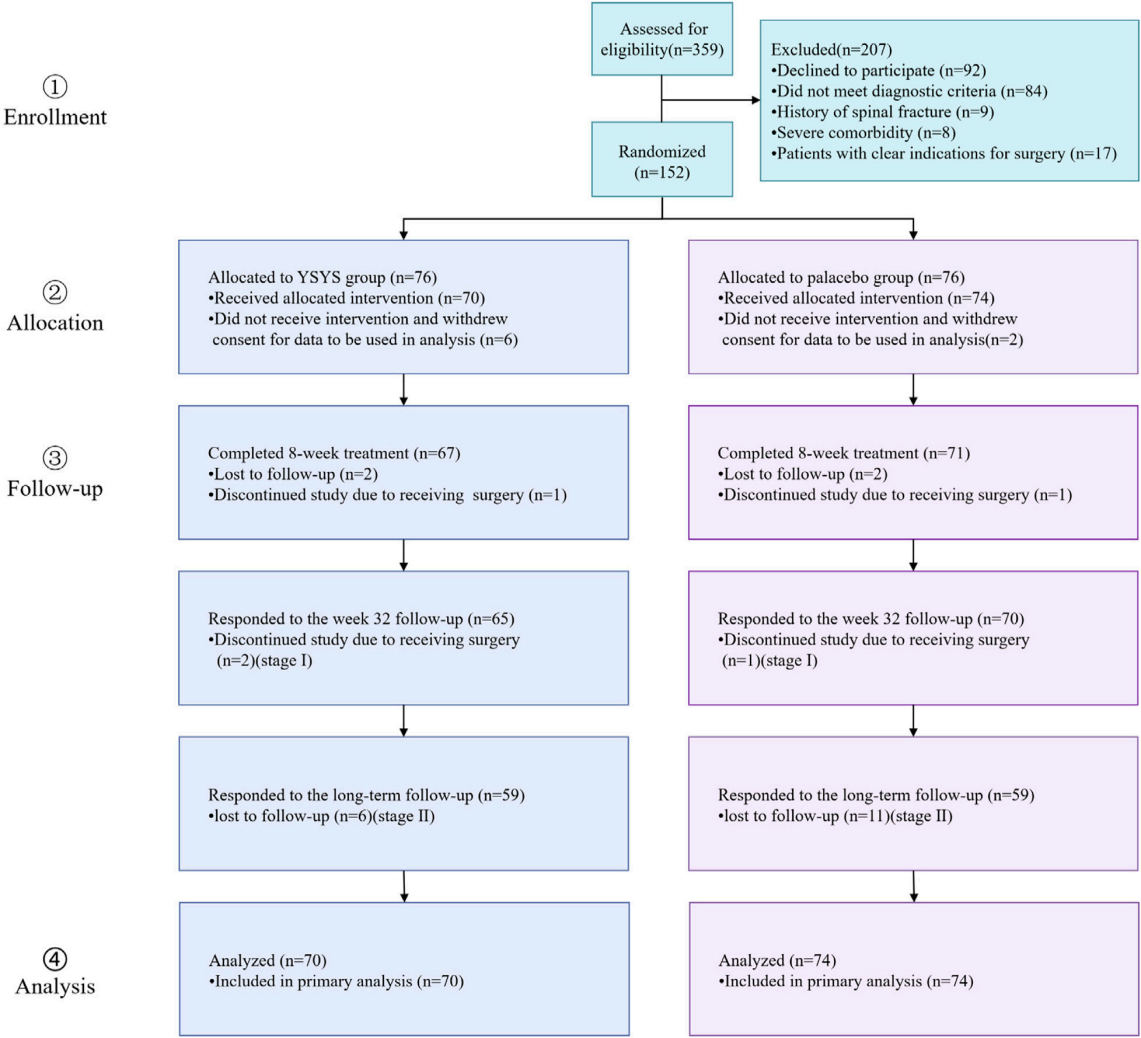
Flow diagram of progress through trial. Abbreviation: YSYS group, Yishenyangsui granule group.

Participants were recruited between December 2016 and February 2018, with the final follow-up conducted from June 5 to 9 July 2021. A total of 359 participants were screened for eligibility, of whom 152 participants were randomly allocated to either the Yishenyangsui granule group or the placebo group. Among the randomized participants, 144 [mean (SD) age, 56.94 (8.97) years] received at least 1 dose of the study medication (Figure 1). The baseline characteristics were similar between the groups (Table 1).

**TABLE 1:** Participant baseline characteristics.

Characteristics	YSYS group (n = 70)	Placebo group (n = 74)
Age, mean (SD), years	57.67 (8.44)	56.26 (9.46)
Sex, (%)		
Male	39 (55.71)	48 (64.86)
Female	31 (44.29)	22 (35.14)
Body mass index, mean (SD)	24.95 (3.95)	24.51 (3.14)
Course of disease, mean (SD), days	1255.36 (1950.90)	1215.27 (1635.75)
JOA score, mean (SD)	13.19 (0.21)	13.72 (0.20)
NDI score, mean (SD)	13.84 (5.26)	11.57 (4.90)
Severity of DCM^a^, n(%)		
Mild	32 (45.71)	42 (56.76)
Moderate	38 (54.29)	32 (43.24)
Had ever received treatment within 2 weeks^b^, n(%)	14 (20.00)	12 (16.22)
Medication	11 (15.71)	12 (16.22)
Manipulation	5 (7.14)	5 (6.76)
Acupuncture	6 (8.57)	4 (5.41)
Traction	5 (7.14)	3 (4.05)
Physical therapy	7 (10.00)	7 (9.46)
Comorbidities, n(%)		
Cardiovascular and cerebrovascular diseases	2 (2.86)	1 (1.35)
Respiratory diseases	4 (5.71)	2 (2.70)
Hepatobiliary disease	0	2 (2.70)
Urologic diseases	2 (2.86)	0
Metabolic disorders	0	2 (2.70)
Herpes zoster	1 (1.43)	0

Abbreviation: JOA, the Japanese Orthopaedic Association; NDI, Neck Disability Index; DCM, Degenerative Cervical Myelopathy; YSYS, group, Yishenyangsui granule group.

^a^ According to the criteria reported by Moghaddamjou et al. (2020), the severity of DCM, was categorized as mild; JOA, score ≥ 14; moderate, JOA = 9–13; severe, JOA ≤ 8.

^b^ These were the treatments that participants had received within 2 weeks before taking the allocated intervention. Participants who had ever received more than one treatment were documented in every independent treatment he or she received, and the same category of treatment with multiple occurrences in a single participant was defined as 1 treatment. The “Medication” did not include Chinese medicinal botanical drugs.

For the primary outcome, the mean JOA score was 13.19 (95% CI, 12.77–13.60) at baseline and 14.66 (95% CI, 14.21–15.10) at week 8 in the Yishenyangsui granule group and 13.72 (95% CI, 13.31–14.12) at baseline and 14.15 (95% CI, 13.72–14.58) at week 8 in the placebo group. The change in JOA score at week 8 was greater in the Yishenyangsui granule group (1.47; 95% CI, 1.20–1.75) than in the placebo group (0.43; 95% CI, 0.16–0.70) (*P* < 0.001) (Table 2).

**TABLE 2:** Primary/secondary outcomes.

Variable	YSYS group	Placebo group	Difference (95% CI)a	*P* value
Total (n = count)	n = 70	n = 74		
(stage I) mean (95% CI) Primary Outcome				
JOA score at Week 8	14.66 (14.21–15.10)	14.15 (13.72–14.58)		
Change at Week 8	1.47 (1.20–1.75)	0.43 (0.16–0.70)	1.04 (0.65–1.42)	0.000
Secondary Outcomes Change in JOA score				
Week 32	1.31 (1.04–1.59)	0.43 (0.17–0.70)	0.88 (0.50–1.26)	0.000
Change in motor function of upper extremities				
Week 8	0.26 (0.16–0.36)	0.07 (−0.03–0.17)	0.19 (0.05–0.33)	0.009
Week 32	0.23 (0.13–0.33)	0.07 (−0.03–0.17)	0.16 (0.02–0.31)	0.028
Change in motor function of lower extremities				
Week 8	0.46 (0.33–0.58)	0.05 (−0.07–0.18)	0.40 (0.23–0.58)	0.000
Week 32	0.43 (0.31–0.55)	0.04 (−0.08–0.16)	0.39 (0.22–0.56)	0.000
Change in the sensory function of the upper extremities				
Week 8	0.41 (0.30–0.53)	0.15 (0.04–0.26)	0.27 (0.11–0.42)	0.001
Week 32	0.39 (0.27–0.50)	0.14 (0.02–0.25)	0.25 (0.09–0.41)	0.002
Change in NDI score				
Week 8	−2.77 (−3.77 to −1.77)	−1.41 (−2.38 to −0.43)	−1.37 (−2.76 to 0.27)	0.055
Week 32	−2.71 (−3.77 to −1.65)	−1.42 (−2.45 to −0.39)	−1.30 (−2.77 to 0.18)	0.086
Change in reading				
Week 8	−0.47 (−0.63 to −0.31)	−0.20 (−0.36 to −0.04)	−0.27 (−0.50 to −0.04)	0.021
Week 32	−0.50 (−0.67 to −0.33)	−0.23 (−0.40 to −0.06)	−0.27 (−0.51 to −0.03)	0.029
Change in recreation				
Week 8	−0.34 (−0.49 to −0.19)	−0.11 (−0.25 to 0.04)	−0.24 (−0.44 to −0.03)	0.028
Week 32	−0.37 (−0.53 to −0.21)	−0.08 (−0.24 to 0.08)	−0.29 (−0.52 to −0.07)	0.012

**FIGURE 2:**
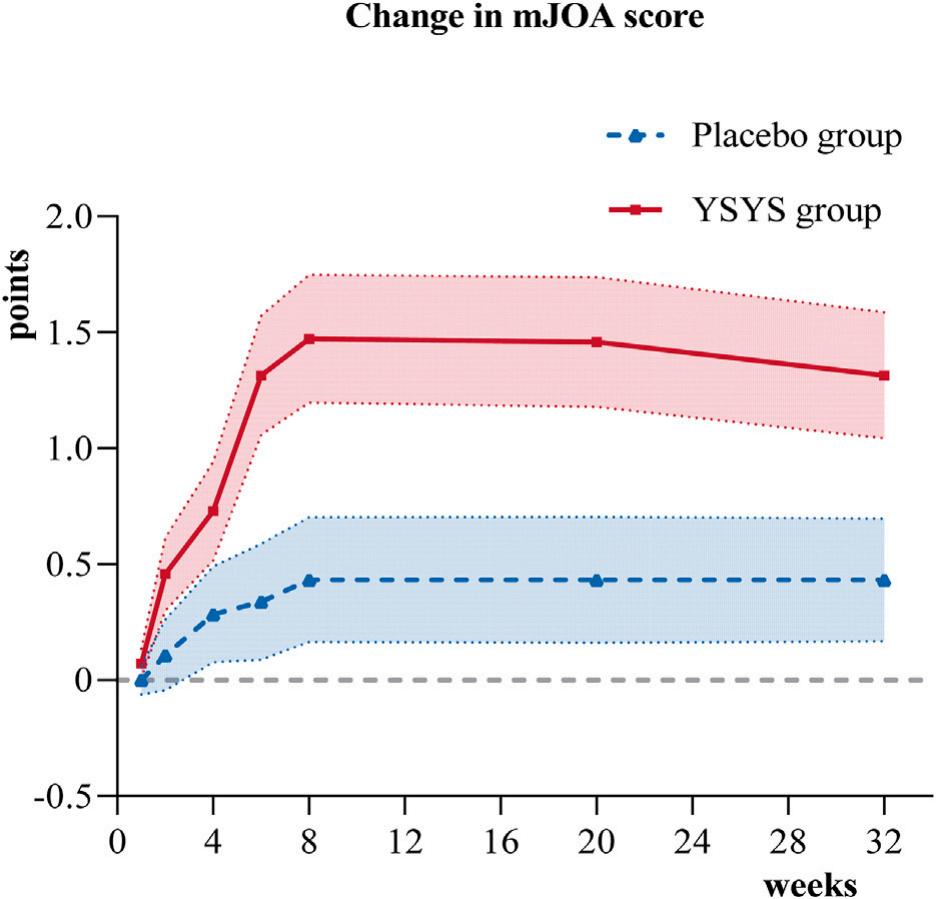
Change in JOA score over time. Abbreviation: YSYS group, Yishenyangsui granule group. The solid red line represents the YSYS group. The Blue dotted line represents the placebo group.

**FIGURE 3:**
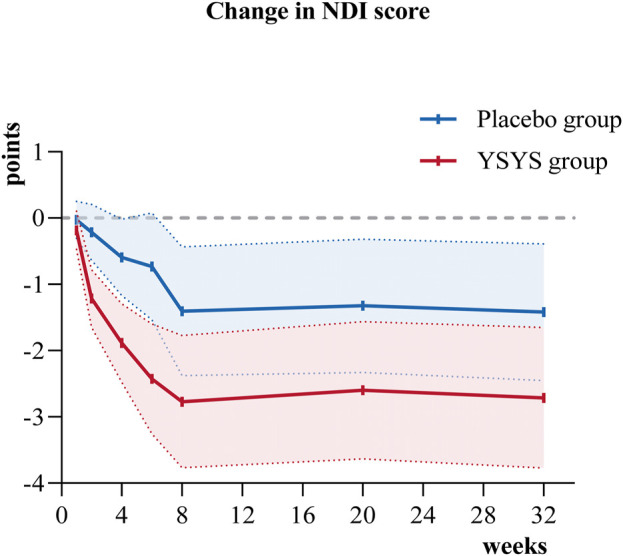
Change in NDI score over time. Abbreviation: YSYS group, Yishenyangsui granule group. The solid red line represents the YSYS group. The solid blue line represents the placebo group.

In terms of the secondary outcomes, similar results were observed in the change in JOA score at week 32. The change in JOA domain scores of motor function in the upper extremities/motor function in the lower extremities/sensory function in the upper extremities in the Yishenyangsui granule group was significantly greater than that in the placebo group (Table 2). The reduction in the reading and recreation domain score in the Yishenyangsui granule group was also significantly greater than that in the placebo group (Table 2). The trend of changes in JOA score and NDI score over time is presented in Figures 2, 3.

**FIGURE 4:**
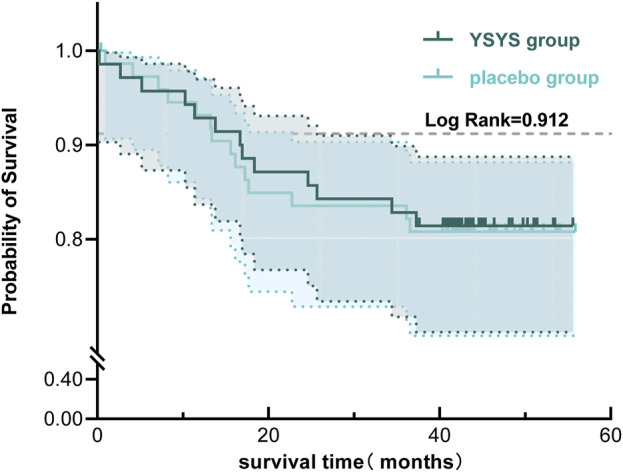
Kaplan-Meier Curve of Time from initiation of the intervention to occurrence of receiving surgical treatment for the two groups. Abbreviation: YSYS group, Yishenyangsui granule group. The solid green line represents the YSYS group. The solid blue line represents the placebo group.

In terms of the long-term outcome (stage Ⅱ), 59 participants in each group participated in the long-term follow-up, with a median follow-up duration of 43.60 months (95% CI, 42.894–44.306). Twenty-five (43.10%) patients in the Yishenyangsui granule group required retreatment, which was less than the 40 (68.97%) patients requiring retreatment in the placebo group (Table 3). There was no significant difference in the patient-reported condition between the two groups (Table 3). The proportions of patients who underwent surgical treatment in the Yishenyangsui granule group and the placebo group were 22.03% (13/59) and 23.73% (14/59), respectively. The mean time from intervention initiation to surgical treatment in the Yishenyangsui granule group [48.373 months (95% CI, 44.674–52.092)] was longer than that of the placebo group [48.177 months (95% CI, 44.417–51.937)] (Table 3; Figure 4).

**TABLE 3:** Long-term outcomes.

Variable	YSYS group	Placebo group	Difference (95% CI)^a^	*P* value
Long-term outcomes (stage Ⅱ)^b^				
Re-treatment data^c^				0.033^e^
No./total No. ^d^				
Out-patient	10/58	16/58		
In-patient (non-operation)	2/58	6/58		
In-patient (operation)	12/58	13/58		
In-/Out-patient	1/58	5/58		
Patient-reported conditions ^f^				0.538^e^
No./total No. ^g^				
Being improved	18/59	10/59		
Remaining stable (non-operation)	22/59	28/59		
Remaining stable (operation)	4/59	4/59		
Getting worse (non-operation)	6/59	7/59		
Getting worse (operation)	9/59	10/59		
Surgical treatment data ^h^				0.912^i^
No./Total No.				
Minimally invasive operation	0/59	2/59		
Open operation	13/59	12/59		
Survival time mean (95% CI), months	48.373 (44.674–52.092)	48.177 (44.417–51.937)		

Abbreviation: YSYS, group, Yishenyangsui granule group.

^a^ The mean differences were calculated using the simple effect analysis based on the repeated measures analysis.

^b^ Median follow-up duration: 43.60 months (95%CI, 42.894–44.306). The shortest follow-up duration was 40.30 months and the longest follow-up duration was 55.87 months. According to the guideline developed by Fehlings et al. (2017), the duration of a long-term follow-up was described to be more than 36 months.

^c^ Re-treatment data was reported by participants and categorized into 4 categories. The same category of re-treatment with multiple occurrences in a single participant was defined as 1 re-treatment.

^d^ Re-treatment data was collected from the completion of the allocated intervention to the long-term follow-up and 58 participants in each group contributed to that data.

^e^ Categorical variables were analyzed using Fisher’s exact probability test.

^f^ Patient-reported condition evaluation was obtained directly from participants at the long-term follow-up either regarding the current status of the patients who had not gone through surgery or regarding the condition before the surgery of the patients who had received the surgery by telephone interview.

^g^ Patient-reported condition was collected at the long-term follow-up and 59 participants in each group contributed to that data.

^h^ Surgical treatment data was reported by participants and collected from the initiation of allocated intervention to the long-term follow-up and 59 participants in each group contributed to that data.

^i^ Log-rank test.

During treatment, adverse events occurred in 4 patients in the placebo group and 11 patients in the Yishenyangsui granule group, mainly mild adverse events such as cold, pharyngitis, cough, and diarrhea, which were categorized as either not related (12 cases) or unlikely related (3 cases) to ingestion of medication by the researchers. All of the adverse events occurred or disappeared in a short period. No participant discontinued treatment or withdrew due to adverse events.

## 4 Discussion

Traditional Chinese Medicine concepts of “deficiency” and “stasis” match modern medical treatments for degenerative cervical myelopathy, showing that neurodegeneration and impaired circulation are related. “Deficiency,” notably in Qi and Kidney essence, refers to neuronal vitality and functional degradation (Du et al., 2020; Li et al., 2015), while “stasis” refers to blood stasis and decreased microcirculation, which modern medicine links to inflammation, oxidative stress, and tissue healing (Xia et al., 2021; Delgado-Montero et al., 2020). These ideas combine TCM philosophy with modern biological treatments for this illness to promote neuron regeneration, reduce inflammation, and improve blood circulation. Over the 8-week treatment period, the Yishenyangsui granules produced greater improvement in the JOA score and greater improvement in retreatment data for the long-term follow-up than the placebo. The effects continued for another 24 weeks after treatment. The incidence rate of adverse events was low.

The JOA scale was used to evaluate neurological function directly. In this study, the Yishenyangsui granule group revealed a better JOA score improvement from the initial visit to the endpoint, with an initial score of 13.19 (95% CI, 12.77–13.60) and an endpoint score of 14.66 (95% CI, 14.21–15.10). In a prospective cohort study of DCM patients who exhibited a decreased JOA score from 14.5 (SD, 1.3) at the initial visit to 14.1 (SD, 2.2) (Sumi et al., 2012) at the endpoint after 6–14 years of follow-up, the patients initially received in-bed Good Samaritan cervical traction for 8 h a day over 2 weeks and then were given ongoing details about their pathological condition every 3–6 months before being advised to take precautions to prevent aggravation of their myelopathy to avoid the need for surgical intervention. Other studies on DCM patients who were treated conservatively showed a JOA score improvement from 14.3 (SD, 1.3) to 14.5 (SD, 1.8) after a 2-year follow-up (Kadaňka et al., 2000) and a further increase to 15.0 (95% CI, 12.2–18.0) after a 10-year follow-up (Kadaňka et al., 2011). The curve in Figure 2 indicates that the Yishenyangsui granule began to play a role from week 2, and the improvement effect reached a maximum at week 8.

The long-term follow-up outcomes in this trial are indicative of the “natural progression” of DCM, which is defined in the literature (Matz et al., 2009) as progression with no treatment or with only nonoperative therapy. The retreatment rate after recurrence in the Yishenyangsui granule group (43.10%) was less than that in the placebo group (68.97%). For patient-reported conditions, this paper revealed that the average proportion of subjects showing improvement in the two groups was 23.73% (28/118), and the proportion of subjects remaining stable and showing deterioration was 49.15% (58/118) and 27.12% (32/118), respectively, which were slightly different from the results reported in the literature. A prospective study with a 3-year follow-up indicated that more than 80% of the patients remained stable after nonsurgical treatment (Tetreault et al., 2015; Shimomura et al., 2007). Roberts (Roberts, 1966; Choi, 2020) reported that one-third of DCM patients who underwent collar immobilization showed improvement, while one-third of them did not experience any improvement, and the remaining one-third experienced a worsened motor deficit at 6.5 years after treatment. Professor Kadaňka (Kadaňka et al., 2011) believes that while the natural progression of DCM is unclear at present, the trend is that an equal proportion of patients may either improve, remain stable, or worsen. The average incidence rate of surgical treatment in the two groups in this study was 22.88% (27/118), and the minimum follow-up duration was 40.30 months. This was roughly the same as reports in the literature in that approximately 20% of patients receiving nonsurgical treatment had to undergo surgical treatment during the follow-up (Zileli et al., 2019), which was lower than the 23%–54% previously reported in the systematic review (Ghobrial and Harrop, 2015). The lack of progress in improving surgical treatment outcomes and patient-reported conditions may be due to patients stopping the administration of granules after 8 weeks. Furthermore, DCM is characterized by a wide range of causes, severity levels, and natural progression in spinal cord damage. In addition to this intricate nature, there is a lack of first indicators and dependable biomarkers that can determine the extent of damage to cerebral tissue, which is crucial for designing therapeutic trials. Therefore, further research is needed to determine whether it is better if the patients take the granule at regular intervals for a longer period.

The mechanism of TCM in the treatment of DCM is complex (Choi, 2020; Ulndreaj et al., 2022; McCormick et al., 2020). Network pharmacology research (Baoyu et al., 2020) indicated that the key targets of the Yishenyangsui granule in the treatment of DCM were STAT3, AKT1, JUN, MAPK1, IL-6, and RELA. By studying the course of chronic spinal cord compression in experimental rat models, it was found that Yishenyangsui granule had a good therapeutic effect (Bowen et al., 2022), and the mechanism might be related to promoting the repair of damaged neurons, regeneration of local spinal microvessels, improving microcirculation disturbance (Bin et al., 2022), increasing the expression of BDNF, and reducing the apoptosis of damaged neurons by inhibiting the expression of key proteins in the Fas/fasl-caspase-8/-3 signaling pathway (Dian et al., 2024). In addition, a large number of basic studies have shown that the main metabolites of the YSYS group can improve spinal cord injury. Tanshinone IIA is acknowledged as a neuroprotective drug that markedly diminishes peroxidation-induced cellular death at the molecular level (Sherawat and Mehan, 2023; Jia et al., 2023; Luo et al., 2022). Yin et al. (2012) used tanshinone to improve microcirculation, thereby exerting a neuroprotective effect. Lin et al. (2020) confirmed that astragaloside inhibits neuronal apoptosis and promotes spinal cord injury repair. Our prior animal studies have also shown that the YSYS group enhanced neurological function and decreased neuroinflammation in rats with spinal cord injury (Supplementary Figures S2, S3). The long-term follow-up results corroborate these findings, demonstrating that YSYS Granules substantially enhance long-term neurological function and diminish the retreatment rate, likely attributable to its neuroprotective, anti-inflammatory, and vascular regeneration processes.

Further research is required to elucidate the mechanism of Yishenyangsui granules. Consequently, subsequent studies should prioritize experimental validation to furnish more definitive proof regarding the efficacy of the YSYS group in the treatment of DCM. While the randomized, double-blind, placebo-controlled experiment can demonstrate the efficacy of the YSYS group in treating DCM, it cannot completely capture their pharmacological and biological actions within the human body. Consequently, additional studies should investigate the precise mechanisms of action of the primary active constituents of the YSYS group in DCM treatment, along with the potential adverse effects associated with their application.

## 5 Conclusion

The YSYS group demonstrated efficacy in the treatment of mild and moderate DCM. The YSYS group consists of many herbal components, some of which may exert numerous effects on the development and progression of DCM. In this randomized, double-blind, placebo-controlled trial, the YSYS group demonstrated efficacy in treating mild to moderate DCM. It also showed enhancements in the retreatment rate and neurological function compared to the placebo group. Despite the lack of statistical significance in enhancing surgical treatment outcomes and patient-reported conditions, the YSYS group seems to be a viable option for the treatment of mild to moderate DCM. Additional research including bigger cohorts and patients with varying degrees of severity may be beneficial in determining the efficacy of the YSYS group in DCM pathology.

## Data Availability

The original contributions presented in the study are included in the article/Supplementary Material, further inquiries can be directed to the corresponding authors.

## Abbreviations

JOA, Japanese Orthopaedic Association; NDI, Neck Disability Index; DCM, Degenerative Cervical Myelopathy; YSYS group, Yishenyangsui granule group; BDNF, Brain-derived neurotrophic factor; STAT3, Signal Transducer And Activator of Transcription 3; AKT1, Alpha serine/threonine-protein kinase; MAPK1, Mitogen-activated protein kinase 1; IL-6, Interleukin 6.
