# Supplementary material for: Yishenyangsui granule for degenerative cervical myelopathy: a randomized, double-blind, placebo-controlled trial with long-term follow-up
Source: Front Pharmacol. 2025 Jan 31;16:1542231. doi: 10.3389/fphar.2025.1542231 (PMC11825510; doi:10.3389/fphar.2025.1542231)
Supplement: Supplementary file 1 [file DataSheet1.pdf]

Supplementary Material

Supplementary Table S1 Detailed information about the YSYS group

Pharmaceutical name	Botanical name	Family and plant parts used	English name	Chinese name	Amount in preparation (g)
Morindae Officinalis Radix	*Gynochthodes officinalis* (F.C.How) Razafim. & B.Bremer	Rubiaceae; Root	Medicinal Morinda Root	Ba Ji Tian	9
Rehmanniae Radix	*Rehmannia glutinosa* (Gaertn.) DC.	Scrophulariaceae; Root tuber	Adhesive Rehmannia Root Tuber	Shu Di Huang	12
Cornu Cervi Degelatinatum	Cervus nippon Temminck	Cervidae; Residue of cornu cervi extracted by water	Degelatined deer-horn	Lu Jiao Shuang	12
Paeoniae Radix Alba	*Paeonia lactiflora* Pall.	Ranunculaceae; Root	White Peony Root	Bai Shao	12
Astragali Radix	*Astragalus membranaceus* Fisch. ex Bunge	Fabaceae; Root	Mongolian Milkvetch Root Membranous Milkvetch Root	Huang Qi	15
Cinnamomi Ramulus	*Cinnamomum burmanni* (Nees & T.Nees) Blume	Lauraceae; Burgeon	Cassiabarktree Twig	Gui Zhi	6
Salviae Miltiorrhizae Radix et Rhizoma	*Salvia miltiorrhiza var. miltiorrhiza*	Lamiaceae; Root and rhizome	Dan - Shen Root	Dan Shen	9
Euonymus alatus	*Euonymus alatus* (Thunb.) Siebold	Celastraceae; branch or appendage	Winged euonymus	Gui Jian Yu	12
Notopterygii Rhizoma et Radix	*Notopterygium incisum* Ting ex H.T.Chang	Apiaceae; Root and rhizome	Incised Notopterygium Rhizome and Root	Qiang Huo	6

Note: Information of "Lu Jiao Shuang" refers to Wang et al(1). The sources of others' information are from *Chinese Medicinal Material Images Database* (https://library.hkbu.edu.hk/electronic/libdbs/mmd/) and *Medicinal Plant Images Database* (https://library.hkbu.edu.hk/electronic/libdbs/mpd/).

The HPLC test method

Determination of 9 main components in the YSYS group (martynoside, notopterol, astragaloside IV, alatamine, nervose, cinnamaldehyde, proline, paeoniflorin, and tanshinone II A) by LC-MS/MS

1. Sample pretreatment method: accurate water decoction (2 ml), addition of methanol (8 ml), high-speed mixing to extract (3 min), extract obtained (1 ml), 14000 rpm speed centrifugation (10 min), supernatant obtained.
2. Instrument: A Waters Acquity-Class ultrahigh-performance liquid chromatography (UHPLC) tandem Waters Synapt G2-SiQ-TOF high-resolution mass spectrometry system was used, using high-purity nitrogen as the atomization gas and high-purity argon as the collision gas.
3. Liquid phase method: The chromatographic column was a UPLC HSS T3 column (100 × 2.1 mm, 1.8 μm), the column temperature was 35 °C, and the injection volume was 10 μL. Gradient elution, mobile phase: 0.1% formic acid water as mobile phase A, 0.1% formic acid acetonitrile as mobile phase B. Flow rate 0.25 mL/min. Ultraviolet detection wavelength: 210 nm-400 nm full-wavelength scanning
4. Mass spectrometry: In positive mode, the positive ESI mode was detected in the range of m/z 50-1500, the scanning mode was MSe, the capillary voltage was set to 2.0 kV for negative ionization mode scanning, the cone hole voltage was 20 V, the source temperature and desolvation temperature were adjusted to 125 °C and 500 °C, respectively, and the cone hole gas flow rate was adjusted to 50 L/H and 800 L/H, respectively. In the positive mode, the detection was carried out in the negative ESI mode in the range of m/z 50-1500, the scanning mode was MSe, the capillary voltage was set to 2.5 kV for neutral ionization mode, the taphole voltage was 20 V, the source temperature and desolvation temperature were set to 125 °C and 400 °C, respectively, and the flow rates of cone gas and desolvent gas were adjusted to 50 L/H and 800 L/H, respectively.
5. Data analysis: The original data collected were processed by Waterworld's UNIFI data processing

software, characteristic peaks were identified, isotope distribution fitting was performed, and element composition matching was performed. Based on the database of 6400 active compounds of traditional Chinese medicine in UNIFI software, key information such as high-resolution molecular ion peaks and fragment ions of characteristic peaks is searched and matched. UniFI software automatically matches the best. Reliable identification results for compounds were obtained.

6. Results: Nine main components were qualitatively analyzed by LC-MS/MS/MS. Nine main components (martynoside, notopterol, astragaloside IV, alatamine, nistose, cinnamaldehyde, proline, paeoniflorin, and tanshinone II A) were detected in the YSYS group.

Supplementary Table S2 Detailed information on UHPLC

Time（min）	A%	B%
0	100	0
3	100	0
12	80	20
70	0	100
76	0	100

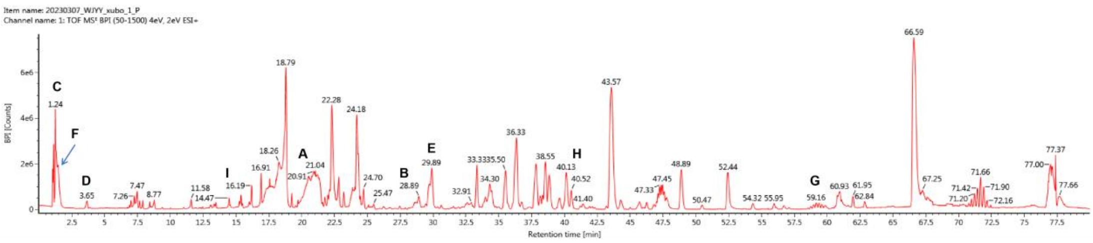

Fig. S1 Ultrahigh-performance liquid chromatography of YishenYangshui granule.

A: cinnamaldehyde, B: Notopterol, C: proline, D: nistose, E: astragaloside, F Alatamine, G: Martynoside, H: tanshinone II A, I: paeoniflorin

Supplementary Table S3 Traditional Chinese medicine terminology and explanation

Term	Definition(2)
Deficiency (虚)	Refers to a lack of vital substances such as Qi, blood, Yin, or Yang in the body, leading to functional decline, including diminished neural activity and regeneration.
Stasis (瘀)	Describes the stagnation of blood or fluids in the body, which can result in poor circulation, inflammation, and tissue damage, aligning with impaired microcirculation in modern medicine.
Traditional Chinese Medicine (TCM)	A holistic medical system developed in China, based on theories like Yin-Yang, Qi, and the Five Elements, utilizing botanical drugs, acupuncture, and other therapies.
Chinese Medicinal Herbs	Refers to plant-based substances used in TCM, which are believed to balance the body’s energy and treat various diseases by improving bodily functions and promoting healing.
Botanical Drug	Plant-derived substances that are processed and standardized for therapeutic use, particularly in the treatment of specific medical conditions as defined by pharmacopeias.

Animal studies of YSYS group

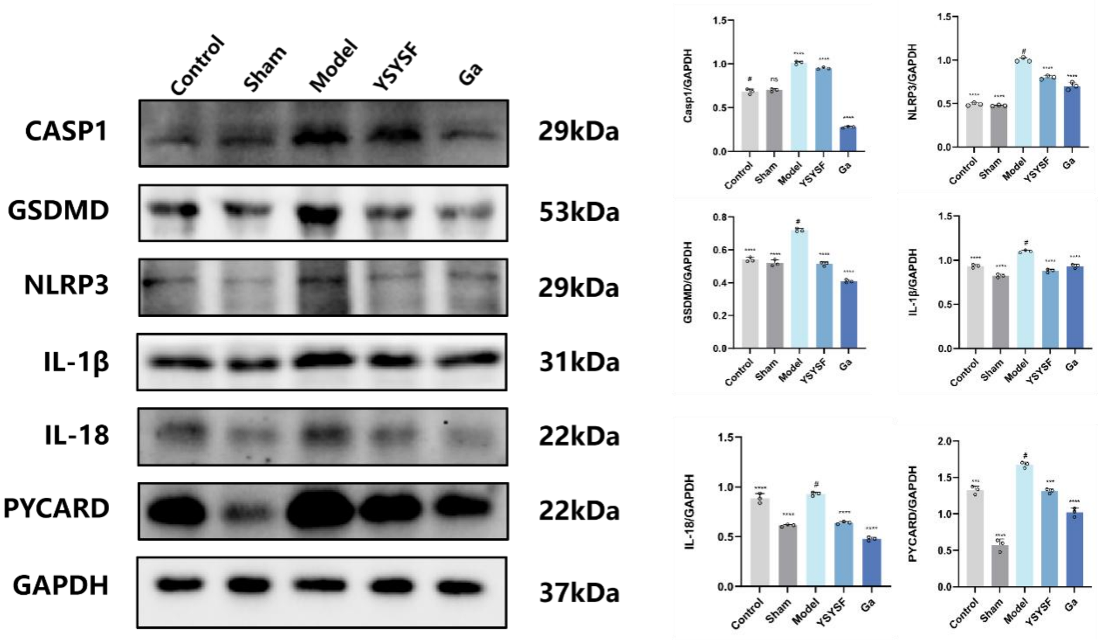

Fig. S2 YSYS group decreased neuroinflammation in rats with spinal cord injury

The Yishen Yangsui formula has been found to successfully suppress the activation of inflammasomes NLRP3 and PYCARD, hinder the release of inflammatory factors IL-1β and IL-18, decrease the expression of pyroptosis-related proteins Caspase1 and GSDMD, and enhance the neurological function recovery in rats with spinal cord injury.

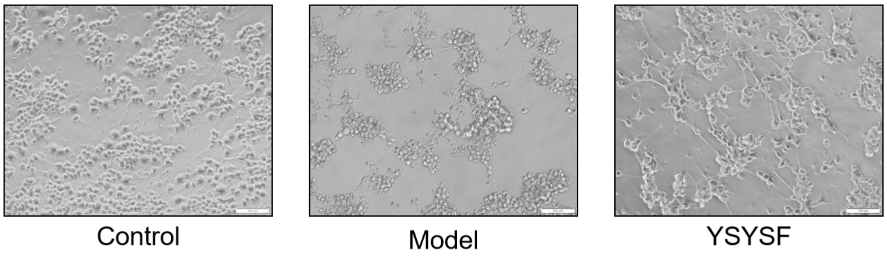

Fig. S3 Morphology of cells in each group under the light microscope (X200)

Under a light microscope, the control group has more cells, complete morphology, and distinct, tightly packed synapses. Cellular pyknosis, synaptolysis, cell clumping, and poor condition are common in the model group. Rat morphology improves significantly in the Yishen Yangsui prescription incorporating serum intervention group compared to the model group. Axons are well-defined and infrequently reduced, with uncommon suspended aggregation.

Reference

1. Wang Y, Sun W, Chen L, Xu X, Wu Y, Zhang J, et al. Anti-Arthritic Activity of Fu-Fang-Lu-Jiao-Shuang on Collagen-Induced Arthritis in Balb/C Mice and Its Underlying Mechanisms. *Pharmacogn Mag* (2015) 11(42):242-9. doi: 10.4103/0973-1296.153065.

2. Lozano F. Basic Theories of Traditional Chinese Medicine. In: Lin Y-C, Hsu ES-Z, editors. *Acupuncture for Pain Management*. New York, NY: Springer New York (2014). p. 13-43.
